# Are the poor differentially benefiting from provision of priority public health services? A benefit incidence analysis in Nigeria

**DOI:** 10.1186/1475-9276-11-70

**Published:** 2012-11-16

**Authors:** Obinna Onwujekwe, Kara Hanson, Benjamin Uzochukwu

**Affiliations:** 1 Health Policy Research Group, Department of Pharmacology and Therapeutics, College of Medicine, University of Nigeria Enugu-Campus, PMB 01129, Enugu, Nigeria; 2 Department of Health Administration and Management, University of Nigeria Enugu-Campus, Enugu, Nigeria; 3 Department of Global Health and Development, London School of Hygiene and Tropical Medicine, London, United Kingdom; 4 Department of Community Medicine, College of Medicine, University of Nigeria Enugu-Campus, Enugu, Nigeria

**Keywords:** Benefit-incidence- analysis, Public health services, BIA, Equity, Nigeria

## Abstract

**Background:**

The paper presents evidence about the distribution of the benefits of public expenditures on a subset of priority public health services that are supposed to be provided free of charge in the public sector, using the framework of benefit incidence analysis.

**Methods:**

The study took place in 2 rural and 2 urban Local Government Areas from Enugu and Anambra states, southeast Nigeria. A questionnaire was used to collect data on use of the priority public health services by all individuals in the households (n=22,169). The level of use was disaggregated by socio-economic status (SES), rural-urban location and gender. Benefits were valued using the cost of providing the service. Net benefit incidence was calculated by subtracting payments made for services from the value of benefits.

**Results:**

The results showed that 3,281 (14.8%) individuals consumed wholly free services. There was a greater consumption of most free services by rural dwellers, females and those from poorer SES quintiles (but not for insecticide-treated nets and ante-natal care services). High levels of payment were observed for immunisation services, insecticide-treated nets, anti-malarial medicines, antenatal care and childbirth services, all of which are supposed to be provided for free. The net benefits were significantly higher for the rural residents, males and the poor compared to the urban residents, females and better-off quintiles.

**Conclusion:**

It is concluded that coverage of all of these priority public health services fell well below target levels, but the poorer quintiles and rural residents that are in greater need received more benefits, although not so for females. Payments for services that are supposed to be delivered free of charge suggests that there may have been illegal payments which probably hindered access to the public health services.

## Introduction

Increasing public health expenditure does not automatically translate into better outcomes for all population groups if the expenditures are not equitably distributed. In Nigeria and many sub-Saharan African countries, skewed resource allocation towards urban-based hospital services, and services that tend to be used by the better-off, have often hindered efforts to improve health outcomes as the additional public spending does not reach those most in need. The money spent gets thinly spread amongst the population segments that need subsidies the most. Greater levels of public spending on public health services could significantly decrease mortality, as demonstrated in the developed world [1].

National health financing systems need to be pro-poor if healthcare targets are to be met. Such systems should therefore incorporate three important dimensions: they should ensure that contributions to costs of healthcare are in proportion to different households’ ability to pay; protect the poor from financial shocks associated with severe illness; and enhance the accessibility of services to the poor [2]. Such systems can only be achieved if healthcare planners are well-informed about the distribution of the benefits of public subsidies and of the burden of paying for health services.

The level of coverage of the population with priority health services in Nigeria has remained low over the years. The 2008 National Demographic Health Survey (NDHS) in Nigeria showed that only 23% of children are fully immunized by age 12–23 months, whilst 8% of households owned at least one insecticide-treated mosquito net, 58% of pregnant women received antenatal care (ANC) from a skilled provider, and only 35% of births take place in a health facility [3]. The NDHS data were disaggregated into different population groupings to provide evidence of equity or inequity, and show evidence of an inequitable distribution of utilisation for most services such as immunisation in children, contraceptives, treatment of acute respiratory infections, malaria and diarrhoea, and ownership of insecticide-treated nets (ITNs).

However, existing data in Nigeria do not link service utilization to the value of benefits received or to payments that are made for different interventions by different population groups. There are concerns that public health interventions, which may not be reaching poor and marginalised populations have amongst others things led investigators to examine the coverage of public health interventions among persons with differing socioeconomic status [4]. This information is required by decision-makers to implement strategies that will increase equity in consumption of public expenditures on priority public health services.

Benefit incidence analysis (BIA) is a powerful tool for assessing how efficiently public spending is targeted to the poor and who benefits from expenditure on public services such as education and health. According to some authors, [5], BIA became an established approach through the work of [6] on Malaysia and that of Selowsky [7] on Colombia. Recipients are usually distinguished by their relative economic position, but the geographic distribution of spending can also be examined, as can the distribution across characteristics such as ethnicity and age [8]. Analysing benefit incidence of public sector expenditure is tantamount to testing fiscal policy performance with respect to reduced poverty and inequality.

The objective of this study was to generate new knowledge about the beneficiaries of government expenditures on priority public health interventions, by evaluating the benefit incidence (based on socio-economic groups, gender, rural–urban location) of a set of publicly-financed public health interventions that are supposed to be provided free of charge.

## Research methods

### Conceptual framework

In this study, BIA focused on a limited set of public services that are supposed to be provided free of charge. Conducting a BIA follows a number of key steps [5,8-10]: (1) Identification of users on the basis of household surveys; (2) Aggregation of users into groups of interest (commonly defined by income levels, region, urban/rural location, poor/non-poor, occupation, ethnicity, etc.); (3) Estimation of the value of the benefit: typically estimated as the cost of providing the service; (4) Accounting for out-of-pocket expenditures by households to access the services; and (5) Examination of the distribution of net subsidies for different population groups. BIA requires: (1) individual or household-level data from household surveys on welfare and on the use of service and receipt of public spending; and (2) information on unit costs of public expenditure to estimate the value of the benefits. Analysis is usually undertaken at the individual level.

### Research area

The research was undertaken in 4 selected Local Government Areas (LGAs); 1 rural and 1 urban LGAs from Enugu and Anambra states respectively (2 LGAs per state). The two state capitals were selected as the urban LGAs and two rural LGAs were selected where it was believed that all the major financing mechanisms were operational.

Enugu is the capital city of Enugu state. There are 17 LGAs in the state, of which 5 are largely urban. Enugu state has an estimated population of about 3,257,198 (from the 2006 Nigerian census). Anambra state has a population of 4,177,828 (from the 2006 Nigerian census). Its capital city is Awka, and it is made up of 21 LGAs, 6 of which are urban. Each state capital has a tertiary hospital and each urban LGA has a public general hospital. There are public health centres in all rural LGAs. The private sector is represented by: private hospitals, clinics, pharmacies, PMDs and mission hospitals, all of which are found in both states.

### Study design and data collection

A pre-tested questionnaire was administered by trained field workers to randomly selected householders from 4 LGAs (1,200 people per LGA). The sample size per state was estimated using: the estimated number of households in the urban and rural LGAs per state; a power of 80% and 95% confidence level to detect socio-economic and other population group differences in consumption of benefits priority public health services. In each selected household, one woman (the primary care giver) – or in her absence the male head of the household -- was interviewed and they provided information about all the household members. A one month recall period was used to collect information on outpatient healthcare expenditure and expenditure on other goods and services.

The questionnaire inquired about the number of individuals living within different households that consumed some priority entirely free health services within six months to the date of interview. The number of times that an individual consumed a free service was also elicited. The questionnaire also inquired about the number of individuals living within households that consumed the above listed public health services within six months to the date of the interview, but that paid some money in order to consume the services. Data was therefore collected on the amounts of money that were paid to consume the listed public health services. Data was also collected on socio-economic and demographic characteristics of the respondents and their households.

The priority public health services that were supposed to be provided free of charge and that were examined in this study were: immunisation services, insecticide-treated nets (ITNs), artemisinin-based combination therapy (ACT) for children and pregnant women; ante-natal care (ANC) in primary health care (PHC) centres; normal delivery in PHC centres; antiretroviral drugs in public facilities; family planning (FP) services; and treatment of tuberculosis.

### Data analysis

Individual-level data were used for the service-specific BIA. The data set for the households reports on 4,873 households, whilst the data for individuals reports on 22,169 people. The frequency distributions of the variables by SES, rural–urban location, and gender were calculated and chi-squared (Chi^2^) tests of trend were used to test for differences in distributions across socioeconomic groups. The Kruskal-Wallis non-parametric test, which reports a Chi^2^ statistic, was used to compare differences in means of continuous variables. The Kruskal-Wallis is the non-parametric equivalent of ANOVA.

Principal components analysis (PCA) was used to create a socio-economic status (SES) index using information on the households’ ownership of a: radio; bicycle; motorcycle; car; refrigerator; generator; kerosene lamp; together with the weekly household cost of food (a good measure of household SES since households typically spend more than 50% of their income on food [11]. A PCA model was estimated over the complete dataset for the household data. Individuals were assigned the index value of their households and then divided into quintiles. Hence, individuals from the same household all had the same SES index. The index was used to divide the households and individuals into five equal sized SES groups (quintiles). The first principal component of the PCA was used to derive weights for the SES index [12]. The highest weight was given to ownership of a fridge (0.53), followed by ownership of a television (0.50), ownership of a car (0.41), ownership of a generator (0.39), ownership of a radio (0.28), per capita food value (0.20), ownership of a bicycle (−0.15), ownership of a motorcycle (0.08), and ownership of a kerosene lamp (−0.03). The quintiles were Q1 (most poor); Q2 (very poor); Q3 (poor); Q4 (less poor); and Q5 (least poor). The measure of inequity in household healthcare payments was the concentration index which varies from −1 and +1. A negative sign denotes that the distribution of the variable of interest favours the poor, and if positive, it means that it favours the least poor [13].

The standard methods for BIA were employed: use micro-data to estimate utilisation of the various services; weight the utilisation of different services by their cost in order to arrive at a total “value” of public subsidies (net of payments); and assess the distribution of these subsidies. The unit costs of the services were gathered from several sources: (1) unit standard hospital charges in the two states – collected from Anambra State Ministry of Health [14] and Enugu State Ministry of Health [15]; (2) unit costs/fees for the services as computed by the National Health Insurance Scheme and in the National Strategic Health Development Plan by Federal Ministry of Health [16]; and (3) unit costs calculated from costing data of Ministries of Health, development partners and from literature [17]. The different perspectives for deriving unit costs were used where appropriate. Ideally, a detailed costing of the services work would have been undertaken as part of this research. However, due to a lack of resources (personnel, time and money) to undertake such a huge exercise, the study used existing data as follows:

•*500 Naira for immunisation: Anambra Ministry of Health*[13,14,16]

•*1,000 Naira for insecticide-treated nets: market survey of average price of ITNs*[13-15]

•*1,000 Naira for artemisinin-based combination therapy (ACT): market survey of average price of ACTs;*[13,14]•*2,000 Naira for ante-natal care (ANC) services: average cost;*[13-15]*.*

•*7,500 Naira for normal delivery: average cost;*[13-15]*.*

•*10,000 Naira for antiretroviral drugs: official price of subsidy fixed by the Federal Government of Nigeria*[10]*.*

•*1,000 Naira for FP: market survey of average price;*[13,14]*.*

•*25,000 Naira for treatment of tuberculosis: average cost of treatment;*[13,14]*.*

Note: 120 Naira = US$1.00.

The values of gross benefits for the different services were arrived at by multiplying the unit costs by service utilisation (utilisation rates). This also depended on the number of times an individual consumed a free service. Hence the value of benefit to an individual that consumed a specific free services two times was double that of an individual that consumed the same free service once. The benefits accruing to individuals belonging to the same households were aggregated to determine whether some SES households were capturing more benefits than others (calculating the total value of services accessed by the households belonging to different SES groups). The data were also analysed to show the relative benefits that the different population groups accrued by comparing the shares of service users in each target group to their share in the population.

The net benefits were calculated by subtracting payments made for the services from the gross value of benefits (gross benefits). However, not all individuals that benefited from free services made payments and conversely, not all people that made payments accessed free services. Hence, the aggregate net benefit (adding net benefits from all services) rather than net benefit for individual services gives a better picture of BIA.

The following indicators were calculated: level of use of priority free public health services by individuals; level of use of priority subsidised public health services by individuals; amounts spent on priority free or subsidised health services; unit subsidies for different healthcare services; benefit-incidence ratios of different free priority public health services; amount of benefits accruing to households belonging to different SES groups; amount of money that people paid for supposedly free services; and net benefits.

Ethics approval for the study was obtained from the University of Nigeria Teaching Hospital Ethics Committee. All the respondents gave informed consent before they were interviewed.

## Results

### Socio-economic and demographic characteristics

A total of 2,446 and 2,472 households were interviewed in Enugu and Anambra states, respectively. There were 2,390 rural households and 2,483 urban households. Data were obtained for 11,047 individuals in Enugu state and 11,169 in Anambra state, and for 12,744 and 9,472 urban and rural individuals, respectively. The overall average household size was 5 people, which was the same in the two states but lower in the rural areas. The mean age of the respondents was 41.6 years. The majority of the respondents were female and had some formal education. Household weekly food expenditure was 3,143 Naira (US$26.2) from the combined data. Most of the households owned functional radios and kerosene lamps. Bicycles, motorcycles, cars, and generators were the least commonly owned household assets. The respondents from the urban areas belonged to better-off SES quintiles when compared with those from the rural areas.

### Benefit incidence for different population groups consuming entirely free services

Rural residents consumed slightly more free priority public health services than their share of the population, compared to urban households (Table 1), with 57% of the population consuming approximately 55% of the services. There was very little difference in access of services by socio-economic group, and no difference by gender. Table 2 shows the number of individuals that consumed services at least once, whilst Table 3 shows the value of benefits of all service consumption by the individuals that did, as some individuals reportedly consumed some services more than once.

**Table 1:** Number of individuals that accessed any free priority public health services, by population group

**Population groupings**	**Population group share in total population**	**Number and % of group that accessed free services**
**By urban–rural areas**		
Urban n = 12,745	57.4%	1,798 (14.1%)
Rural n = 9,473	42.6%	1,485 (15.7%)
Chi-square (p-value)		10.6 (.001)
**By gender**		
Males n = 10,069	45.5%	1,498 (14.9%)
Females n = 12,062	54.5%	1,783 (14.8%)
Chi-square (p-value)		.07 (.40)
**By SES**		
Quintile 1 n = 4,437	20.0%	695 (15.7%)
Quintile 2 n = 4,443	20.0%	711 (16.0%)
Quintile 3 n = 4,425	19.9%	632 (14.3%)
Quintile 4 n = 4,431	19.9%	623 (14.1%)
Quintile 5 n = 4,433	20.0%	614 (13.9%)
Chi-square (p-value)		13.8 (.008)
Concentration index (CI)		−0.03

**Table 2:** Number of individuals that accessed specific free public health goods and services, by population group

	**Immunisation**	**Insecticide-treated bets (ITNs)**	**Anti-malaria drugs**	**Ante-natal care (ANC)**	**Child-birth service**	**Anti-retroviral drugs (ARVs)**	**Family Planning services (FP)**	**Treatment of tuberculosis (TB)**
	**n (%)**	**n (%)**	**n (%)**	**n (%)**	**n (%)**	**n (%)**	**n (%)**	**n (%)**
**Utilization by urban-rural residence**								
Urban n = 12,745 (57.4%)	1,649 (55%)	308 (98%)	22 (36%)	16 (73%)	1 (33%)	0 (0%)	0	1 (14%)
Rural n = 9,473 (42.6%)	1,343 (45%)	5 (2%)	39 (54%)	6 (27%)	2 (67%)	1 (100%)	0	6 (86%)
Chi-square (p-value)	45.2 (.0001)	278.9 (.0001)	8.1 (.003)	3.0 (.064)	.55 (.43)	1.2 (.45)	N/A	4.8 (.034)
**Utilization by gender**								
Male n = 10,069 (45.5%)	1,429 (48%)	107 (34%)	21 (34%)	0 (0%)	0 (0%)	0 (0%)	0	3 (43%)
Female n = 12,062 (54.5%)	1,563 (52%)	206 (66%)	40 (66%)	22 (100%)	3 (100%)	1 (100%)	0	4 (57%)
Chi-square (p-value)	60.3 (.0001)	18.6 (.0001)	3.21 (.047)	18.8 (.0001)	2.5 (.16)	.85 (.54)	N/A	.027 (.60)
**Utilization by SES**								
Quintile 1 n = 4,437 (20%)	604 (20.2%)	29 (9.3%)	25 (41%)	2 (9%)	1 (33%)	0 (0%)	0	2 (29%)
Quintile 2 n = 4,443 (20%)	664 (22.2%)	59 (18.9%)	8 (13%)	2 (9%)	0 (0%)	0 (0%)	0	2 (29%)
Quintile 3 n = 4,425 (19.9%)	585 (19.6%)	56 (17.8%)	8 (13%)	1 (5%)	1 (33%)	0 (0%)	0	2 (29%)
Quintile 4 n = 4,431 (19.9%)	569 (19.0%)	94 (30%)	12 (20%)	12 (55%)	1 (33%)	0 (0%)	0	1 (14%)
Quintile 5 n = 4,433 (20%)	570 (19.1%)	75 (24%)	8 (13%)	5 (23%)	0 (0%)	1 (100%)	0	0 (0%)
Chi-square (p-value)	64.6 (.0001)	55.6 (.0001)	15.3 (.005)	15.4 (.04)	2.22 (.73)	4.3 (.37)	N/A	2.8 (.72)
Concentration index (CI)	−0.02	0.17	−0.20	0.27	−0.13	-	-	−0.29
**Total**	2,992	313	61	22	3	1	0	7

n= number of people that used the services.

**Table 3:** Distribution of gross benefits of consumption of specific free public health goods and services, by population group (in Naira)

	**Immunisation**	**ITNs**	**Anti- malaria drugs**	**ANC**	**Childbirth**	**ARVs**	**FP**	**TB**
	**Mean (SD)**	**Mean (SD)**	**Mean (SD)**	**Mean (SD)**	**Mean (SD)**	**Mean (SD)**	**Mean (SD)**	**Mean (SD)**
**Gross benefits by urban-rural residence**								
Urban	457.3 (140.0)	176.2 (381.1)	13.7 (125.8)	81.3 (1,224.2)	4.3 (179.4)	0	0	14.3 (598.5)
Rural	419.7 (183.7)	3.3 (57.5)	33.7 (246.7)	11.0 (181.1)	10.3 (278.4)	0	0	159.8 (2,576.3)
Kruskal-Wallis (p-value)	45.2 (.0001)	278.8 (.0001)	8.2 (.004)	2.9 (.08)	0.47 (.49)	-	-	4.8 (.03)
**Gross benefits by gender**								
Males	463.4 (130.3)	71.9 (258.4)	16.2 (141.5)	NA	NA	0	0	69.1 (1,609.0)
Females	419.7 (183.6)	116.5 (321.1)	28.5 (224.9)	NA	NA	0	0	88.1 (1,914.8)
Kruskal-Wallis (p-value)	60.3 (.0001)	18.6 (.0001)	3.2 (.07)	18.7 (.0001)	2.6 (.10)	-	-	0.02 (.88)
**Gross benefits by SES**								
Quintile 1	398.3 (201.4)	40.9 (198.1)	41.4 (226.3)	8.8 (170.6)	11.0 (287.6)	0	0	76.0 (1,377.3)
Quintile 2	447.9 (152.9)	83.0 (276.1)	12.8 (124.3)	34.4 (535.0)	0	0	0	109.5 (2,134.7)
Quintile 3	447.9 (152.9)	90.8 (287.5)	24.5 (281.9)	55.6 (1,374.4)	12.3 (303.2)	0	0	164.5 (2,865.3)
Quintile 4	450.1 (150.0)	154.1 (361.3)	19.7 (139.0)	102.0 (1,207.9)	12.3 (304.2)	0	0	41.5 (1,018.1)
Quintile 5	460.4 (135.2)	124.2 (330.1)	15.0 (134.5)	53.5 (825.0)	0	0	0	0
Kruskal-Wallis (p-value)	64.5 (.0003)	56.7 (.0001)	15.3 (.004)	20.3 (.0004)	2.0 (.73)	-	-	2.1 (.72)
Concentration index	0.03	0.19	−0.16	0.25	−0.11	-	-	−0.22

Immunisation services were the most commonly used free services, consumed by individuals from 2,992 households (Table 2). They were followed by ITNs (313 people from households) and free antimalarial drugs (people from 61 households). Only one household had one person in the sample that accessed free HIV treatment services. The results also show that people from 22 households consumed free ANC, 3 free deliveries, 7 TB treatments, and 165 used “other” free services. In some of the households, the services were consumed by more than one individual.

Compared to their share in the sample population, rural dwellers accessed relatively more immunisation services, antimalarial drugs, and TB treatment services compared to urban dwellers (Table 2). Conversely, urban residents accessed more of the free ITNs and ANC services. Females generally used more of the free services than males did. Use of immunisation services was higher for poorer SES quintiles, and the poorest SES quintiles benefited relatively more from free antimalarial drugs. However, the better-off quintiles captured the majority of the benefits of ITNs and ANC services.

Table 3 translates the level of utilisation of various services into monetary units, valuing utilisation by the unit cost of the different services and comparing these across the different population groups. The benefits for individuals that utilized free services more than once was higher than those for individuals that utilized free services only once. The mean value per person of benefits of consuming immunisation services was 439.5 Naira (US$3.7). It was 22.9 Naira (US$0.20) for ITNs, 49.4 Naira (US$0.41) for antimalarial drugs, 7.0 Naira (US$0.06) for ANC, and 79.4 Naira (US$0.67) for TB services. Because there was effectively no consumption of family planning and ARVs, the benefits of these services was zero. The distribution of these benefits favoured urban areas for immunisation services, ITNs and ANC, whilst it favoured rural residents for anti-malaria drugs, childbirth services and TB treatment. Males received slightly more of the benefits of immunisation services, whilst females received more of the benefits of all of the other services. The table also shows that the better-off SES quintiles captured more of the benefits of immunisation services, ITNs and ANC services, whilst the poorer SES quintiles captured more of the benefits of free anti-malaria drugs and treatment for TB. The obvious disparity between the utilisation incidence analysis (which showed that use of immunization was more among poorer SES quintiles) and the BIA (which shows that the benefits favoured the better-off SES quintiles) was because more individuals in better-off SES quintiles consumed the services more than once, whilst the poor individuals consumed mostly once.

### Benefit incidence for different population groups consuming supposedly free services but made various payments for them

Although all of these priority services are notionally free, large numbers of people spent money on immunisation services, ITNs, anti-malaria drugs, ANC, child birth services, and TB treatment. However, we conducted no further analysis to determine whether these expenditures were informal payments to public providers or payments made to private providers. This is because for some reason people are choosing to consume these services in the private sector even though they are available in the public sector “for free”.

It was found that 523 (4.1%) and 244 (2.6%) of urban and rural residents respectively made payments in order to consume some of the services. Generally, more urbanites spent money on most public health services except for treatment of TB compared to rural dwellers. For instance, 56% of those who paid something for immunization services were urban residents and 44% were rural residents. Also, 56% of females and 44% of males spent money on the services. It was found that 4.2% of men stated that they paid for ANC, most likely paid for their wives. Those in the lowest SES groups were most likely to make a payment for immunisation services.

Table 4 presents the results of the people that made payments for the supposedly free services. The cases in Table 4 are different from those in Table 2 which refers to only people that consumed free services without having to pay for them. Table 4 illustrates that in terms of their share of total population, the urban dwellers made more payments for anti-malaria drugs and childbirth services, whilst the rural dwellers made more payments for immunisation services, ITNs, ANC and treatment of TB. Females generally made more payments than males compared to their share of the total population. Also, poorest SES quintile consumed and made most payments for immunisation services, whilst the better-off SES quintiles consumed and made most payments for all the other services compared to their share of the total population.

**Table 4:** Users of different supposedly free public health goods and services who made payments for them

	**Immunisation**	**ITNs**	**Anti-malaria drugs**	**ANC**	**Childbirth**	**ARVs**	**FP**	**TB**
	**n (%)**	**n (%)**	**n (%)**	**n (%)**	**n (%)**	**n (%)**	**n (%)**	**n (%)**
**Number that paid by urban-rural residence**								
Urban	193 (55.6%)	0 (0%)	280 (83.6%)	27 (56.3%)	21 (75.0%)	1 (100%)	0	1 (25%)
Rural	154 (44.4%)	4 (100%)	55 (16.4%)	21 (43.7%)	7 (25.0%)	0	0	3 (75%)
Chi-square (p-value)	69.9 (.00001)	20.1 (.001)	.77 (.44)	29.1 (.00001)	2.4 (.17)	.17 (.85)	Na	11.3 (.012)
**Number that paid by gender**								
Male	152 (43.8%)	0	120 (35.8%)	NA	NA	0	0	2 (50%)
Female	195 (56.2%)	4 (100%)	215 (64.2%)	NA	NA	0	0	2 (50%)
Chi-square (p-value)	11.6 (.001)	2.3 (.30)	.26 (.63)			Na	1.7 (.38)	.33 (.63)
**Number that paid by SES**								
Quintile 1	81(23.5%)	1 (25%)	36 (10.8%)	13 (27.1%)	4 (14.3%)	0	0	1 (25%)
Quintile 2	67 (19.4%)	1 (25%)	57 (17.1%)	5 (10.4%)	4 (14.3%)	0	0	2 (50%)
Quintile 3	70 (20.3%)	1 (25%)	64 (19.2%)	6 (12.5%)	5 (17.9%)	0	0	0
Quintile 4	65 (18.8%)	1 (25%)	78 (23.4%)	15 (31.3%)	8 (28.6%)	0	0	0
Quintile 5	62 (18.0%)	0	98 (29.4%)	9 (18.8%)	7 (25.0%)	1/165 (0.6%)	0	1 (25%)
Chi-square (p-value)	31.4 (.00001)	2.0 (.73)	6.3 (.18)	14.8 (.005)	.54 (.97)	2.7 (.61)		5.1 (.28)
Concentration index	−0.02	−0.17	0.03	−0.13	−0.03	-	-	−0.22
Total	347	4	335	48	28	1	0	4

n=number of people that paid for the services.

The mean payment for only the people that paid was 206 Naira (US$ 1.7) for immunisation, 543 Naira (US$4.5) for ITNs, 1071 Naira (US$8.9) for anti-malaria drugs, 2020 Naira (US$16.8) for ANC, 11064 Naira (US$92.2) for childbirth services and 793 Naira (US$6.6) for treatment of tuberculosis. Table 5 shows that in urban areas more money was spent for all services except for child birth services compared to rural areas for the general population. There was also higher expenditure amongst better-off SES quintiles.

**Table 5:** Mean payment for specific public health goods and services, by population group (in Naira)

	**Immunisation**	**ITNs**	**Anti-malaria drugs**	**ANC**	**Child-birth**	**ARVs**	**FP**	**TB**
	**Mean (SD)**	**Mean (SD)**	**Mean (SD)**	**Mean (SD)**	**Mean (SD)**	**Mean (SD)**	**Mean (SD)**	**Mean (SD)**
**Expenditures by urban-rural residence**								
Urban	56.2 (180.5)	0 (0)	613.3 (2,214.2)	71.5 (411.6)	443.6 (3,160.0)	0 (0)	0 (0)	0 (0)
Rural	125.0 (320.9)	22.8 (205.3)	168.1 (407.8)	566.4 (2,518.7)	728.6 (4,779.3)	0 (0)	0 (0)	25.9 (172.6)
Kruskal-Wallis (p-value)	117.2 (.0001)	22.3 (.0001)	8.3 (.004)	30.5 (.0001)	2.6 (0.11)	-	-	17.1 (.0001)
**Expenditures by Gender**								
Male	84.7 (245.6)	0 (0)	371.8 (738.6)	NA	NA	0 (0)	0 (0)	.80 (12.0)
Female	70.1 (221.5)	5.5 (100.9)	630.6 (2,479.2)	NA	NA	0 (0)	0 (0)	5.6 (83.7)
Kruskal-Wallis (p-value)	6.5 (.01)	2.3 (.12)	0.3 (.59)	25.3 (.0001)	16.4 (.0001)	-	-	0.02 (.90)
**Expenditures by SES**								
Quintile 1	73.7 (200.2)	.29 (2.43)	197.05 (441.5)	380.7 (1,379.0)	206.1 (858.1)	0 (0)	0 (0)	2.7 (22.2)
Quintile 2	69.6 (191.7)	.95 (9.8)	373.49 (1,107.8)	48.1 (322.9)	490.2 (3,177.9)	0 (0)	0 (0)	21.0 (160.9)
Quintile 3	108.5 (291.5)	.44 (4.7)	358.25 (691.1)	261.1 (2,135.8)	531.3 (4,312.5)	0 (0)	0 (0)	0 (0)
Quintile 4	56.5 (232.8)	11.83 (153.9)	691.12 (3,275.4)	142.7 (626.7)	573.7 (3,325.0)	0 (0)	0 (0)	0 (0)
Quintile 5	74.5 (222.9)	.00 (.00)	773.7 (1,883.1)	51.5 (278.6)	476.9 (3,735.0)	0 (0)	0 (0)	0 (0)
Kruskal-Wallis (p-value)	19.5 (.0006)	2.0 (0.73)	9.0 (.06)	15.6 (.004)	0.68 (.095)	-	-	7.9 (.09)
Concentration index	−0.10	0.13	0.13	−0.41	−0.08	-	-	−0.45
Total	75.6 (230.7)	3.5 (80.4)	535.7 (2,026.0)	150.97 (1,088.8)	485.8 (3,444.4)	0 (0)	0 (0)	3.9 (67.1)

### Net benefit incidence

Table 6 shows the net benefit to individuals for different services and for aggregate net benefit from all the services to different population groups. It shows that for some of the services, there were negative net benefits, in other words, the amount paid out-of-pocket exceeded the cost of the services. This occurred for consumption of anti-malarial drugs, ANC and childbirth services. All of these involve a lot of private sector provision, where costs may be greater and/or profits are earned. However, immunisation services had positive net befit for all population groups. The net benefit due to immunisation was incurred more for rural dwellers, residents of Anambra state, males and worse-off SES groups. Conversely, net benefit from ITNs was more for urban residents, females and better-off SES groups. The poor received greater aggregate net benefits of priority public healthcare services, with a negative concentration index and aggregate net benefits decreased as SES quintile increased (Table 5). Aggregate net benefit was also higher in rural areas compared to urban areas. There was no statistically significantly difference (p>0.5) in aggregate net benefit between males and females.

**Table 6:** Distribution of net benefits of consumption of specific free public health services by population group (in Naira)

	**Immunisation**	**ITNs**	**Malaria drugs**	**ANC**	**Child-birth**	**ARVs**	**FP**	**TB**	**Cumulative net benefit**
	**Mean (SD)**	**Mean (SD)**	**Mean (SD)**	**Mean (SD)**	**Mean (SD)**	**Mean (SD)**	**Mean (SD)**	**Mean (SD)**	**Mean (SD)**
**Net benefits by urban-rural residence**									
Urban	62.3 (169.4)	24.2 (153.6)	−24.3 (476.2)	8.2 (459.2)	−17.7 (649.6)	0 (0)	0 (0)	2.0 (221.5)	54.7 (1,000.6)
Rural	68.13 (179.0)	0.30 (30.9)	3.23 (109.8)	−4.4 (272.7)	−5.4 (483.7)	0 (0)	0 (0)	23.5 (994.7)	88.7 (1,214.3)
Kruskal-Wallis (p-value)	4.5 (.03)	223.4 (.0001)	106.9 (.0001)	1.65 (.20)	3.5 (.06)	-	-	1.1 (.29)	9.25 (.002)
**Net benefits by gender**									
Male	68.6 (176.9)	10.6 (102.5)	−6.6 (139.0)	NA	NA	0 (0)	0 (0)	9.91 (610.2)	82.6 (675.5)
Female	61.9 (171.1)	16.9 (130.9)	−17.7 (482.9)	NA	NA	0 (0)	0 (0)	12.25 (719.9)	58.2 (1,354.7)
Kruskal-Wallis (p-value)	8.29 (.004)	39.9 (.0001)	41.7 (.0001)	4.22 (.38)	1.1 (0.9)	-	-	−4.4 (1.0)	3.1 (.079)
**Net benefits by SES**									
Quintile 1	65.4 (173.4)	6.3 (79.2)	3.1 (111.4)	−4.8 (191.9)	−1.4 (155.3)	0 (0)	0 (0)	11.2 (530.8)	79.8 (635.2)
Quintile 2	73.1 (180.1)	13.5 (115.6)	−8.0 (194.8)	4.25 (218.3)	−11.3 (485.3)	0 (0)	0 (0)	16.4 (839.9)	88.3 (1,056.1)
Quintile 3	61.8 (173.3)	12.4 (110.6)	−6.3 (169.8)	−6.7 (341.8)	−11.7 (696.3)	0 (0)	0 (0)	22.5 (1061.2)	72.1 (1,365.7)
Quintile 4	62.4 (170.7)	21.0 (148.0)	−25.8 (667.6)	16.2 (684.8)	−20.2 (666.5)	0 (0)	0 (0)	5.6 (375.6)	59.2 (1,259.3)
Quintile 5	61.4 (170.3)	16.9 (129.0)	−26.0 (391.7)	5.3 (308.3)	−17.8 (724.2)	0 (0)	0 (0)	0 (0)	46.6 (1,029.1)
Kruskal-Wallis (p-value)	13.0 (.011)	39.9 (.0001)	41.7 (.0001)	4.22 (.38)	1.1 (.90)	-	-	3.2 (0.52)	16.0 (.003)
Concentration index	−0.02	0.16	0.48	0.90	0.27	-	-	−0.24	−0.11
Total	64.8 (173.6)	14.0 (118.6)	−12.58 (368.0)	2.84 (390.8)	−12.46 (564.69)	0 (0)	0 (0)	11.2 (671.6)	69.2 (1,098.1)

## Discussion

It was surprising to find that very few individuals had consumed wholly free services. This is despite the purported widespread availability in the study area of free immunisation services and malaria treatment services for pregnant women and children under-five. The finding that immunisation services were the most commonly used free service, followed distantly by Insecticide Treated Nets (ITNs) and antimalarial drugs was expected since immunisation of children through the National Programme on Immunisation is implemented on a wide scale in the fight to eradicate polio in the country. Through the availability of free ITNs and artemisinin-based combination therapy (ACTs) through the Global Fund to Fight AIDS, TB and Malaria, these malaria control tools should be widely deployed. However, this study shows that procurement and deployment of the materials by the government and development partners to health facilities and government stores do not mean that they actually reach the people.

A positive finding was that the consumption of antimalarial drugs and tuberculosis (TB) services was pro-poor, and pro-rural. However, the distribution of ITNs and free antenatal care (ANC) both favoured the better off quintiles, and urban populations. A previous study in Nigeria found that urban dwellers received more antimalarial drugs compared to rural dwellers, but rural dwellers owned more ITNs compared to the urbanites [3], which is contrary to the results from this study but the reasons for the disparity are not clear. It is generally accepted that government health expenditures should disproportionately benefit the poor. And yet in most developing countries the opposite is the case [18]. To the extent that need is greater among poor and among rural communities, this reflects an equitable distribution. Some of the results are unlike an analysis in Asia that showed that the distribution of public healthcare is pro-rich in most developing countries [19].

The utilisation that is reported of free services is potentially a mix of public and private utilisation since the questionnaire did not differentiate between the two, and BIA is about the incidence of public subsidies. However, it is almost impossible to find utilisation of free services in the private sector, hence, we can be almost 100% sure that reported utilisation of free services occurred entirely in the public sector and does really represent public subsidies. Nonetheless, a note of caution should still be maintained that the benefits measured could be slightly overestimating actual benefits. However, in the case of immunisation services, the assumption that free services are provided in the public sector are more likely to hold and BIA of consumption of free immunisation services may in actual fact represent the net benefit of public subsidies for such services. It is only in the private sector that people pay to have immunisation. The results showed negative net benefits for those services that are also provided by the private sector: drugs for treatment of malaria, ANC and childbirth services.

It was also found that some people spent money on services that are supposed to be free including, immunisation services, ITNs, anti-malaria drugs, ANC, child birth services, ARV, FP services and treatment of TB. More money was generally spent for all services in the urban areas except for child birth services compared to rural areas. However, compared to their share of the total population, the rural dwellers spent more money on four of the services indicating a greater burden to access public health services on the rural dwellers. There was also more expenditure amongst better-off SES quintiles compared to their share of the total population. The money that was paid for the supposedly free public health services may due to the imposition of formal user charges, private sector use of these services, or some degree of informal charging.

There was no clear underlying reason for the disparity in benefit incidence of the various public health services to different sexes and people living in urban and rural areas. However, the fact that pregnant women receive free ITNs and anti-malarial drugs from public health facilities could have contributed to their capturing higher benefits for those commodities. The finding that compared to their share in the population, rural dwellers marginally consumed more immunisation services and anti-malarial drugs and TB treatment services compared to urban dwellers was reassuring for control of the diseases, because usually people residing in rural areas lack access to healthcare services. However, the finding that urbanites consumed more of free ITNs and ANC services could be as a result of concentration of net distribution outlets and public health facilities in the urban areas.

The fact that the better-off SES consumed more of ITNs and ANC, represents inequity in the deployment of the two essential free services, which should be corrected using appropriate strategies. The 2008 NDHS also reported inequalities in ownership of ITNs, consumption of antimalarial drugs and ANC favouring the better off [3]. Also, the explanation for the greater benefit incidence for treatment of TB by the poorer SES groups is clear since TB is a disease of poverty. However, the reasons for the inequity in the others are less clear, but could be due to the ‘law of inverse equity’ [20,21], where the rich capture more of the benefits of publicly provided services when coverage is low, and that as coverage increases the poor will then start benefiting equally. It could also be due to higher formal and informal political activism by the beneficiaries, as it has been found that individuals with higher political activism have better access to health services than others [22].

The finding that more urbanites spent money on most public health services except for treatment of TB compared to rural dwellers is probably because the services were more available in the urban areas and the urbanites also had more stable disposable income to spend on the health services. There are also higher cost providers in the urban areas. In addition, it is reasonable that females that had more access to services paid more, although it was surprising to discover females paid for services that are free to those that are pregnant. Also, the significant finding that payment for immunisation services increased as SES increased is probably an income effect. People that have more money were more willing to pay to receive the essential services. An implication is that the better-off are getting the services in places they have to pay. When viewed from the point that most of the services have externalities, the negative influences on non-coverage of all the needy people become worrisome. Nonetheless, while the rich are more likely to pay than the poor, the fact that the poor are as likely to use suggests that the immunisation programme is working.

One limitation of the study is that the one-month and six-month (for some priority free services) recall periods may not lead to very accurate collection of data on household health expenditures and consumption of some free services. Also, the questionnaire did not differentiate between public and private service provision and costs because the free services are almost entirely provided by the public sector, but may be delivered by the private sector, thus necessitating strong assumptions about patterns of use and expenditures. For instance, government uses private sector providers to deliver immunisation services in many remote parts of Nigeria by supplying them vaccines and syringes free of charge. was Also, the fact that our survey did not distinguish between pregnant and non-pregnant women beneficiaries in BIA to allow for a more robust conclusion about whether free services for pregnant are really free to them is a study limitation. It did not also examine relative need for services or outcomes. Another possible limitation of the study was we only examined the distribution of benefits for a limited range of public health services. Although the information presented is very useful for programmatic purposes, it may not provide a full set of information required by policymakers to have the full picture about the population groups that benefit from public expenditures. Such comprehensive information will help in holistically ensuring that public expenditures are equitably consumed by different population groups, especially in terms of ensuring vertical equity.

In computing BIA, future studies should investigate use and cost of public services at different levels of health facility, and be able to disaggregate consumption of public subsidies by age-groups and whether women were pregnant or not and identify public sector utilization. This is especially important in case of immunisation services where different vaccines are given at different times and depending on whether or not a woman is pregnant. These will require more specific unit costs to be used in the computation of benefits. It will also be important to understand whether the payments for supposedly free public health services are legal or illegal. Also, future studies should attempt to compute unit costs from private and public sources and use them to value the benefits of services that are delivered through the different sectors. Furthermore, the indirect costs of consumption of services from different providers should be computed to explain further reasons why people would opt for the private sector where they will pay some money instead of the private sector where they may not have to pay any fee to consume the services.

Overall, it was reassuring to find that the poor gained more aggregate net benefits from priority public healthcare services and net benefits decreased as SES quintile increased. This also implies that if the coverage with these services is increased, the poor will benefit more and will be prevented from developing many diseases, most of which lead to their incurring impoverishing catastrophic health expenditures. However, the low and inequitable coverage with public health services, possible illegal payments, that could have further decreased access to the public health services are areas that require programmatic and policy interventions to address. The reasons that were provided by the 2008 National Demographic Health Survey (NDHS) why many children were not receiving immunisations included lack of information, fear of side effects and the venue for immunisation being located too far away [3]. The benefit incidence analysis framework is a useful tool for informing resource allocation decision-making in order to ensure equity of government spending on priority public health services. The government and development partners should develop ways and means of scaling-up the free distribution of vital public health services, whilst developing and implementing strategies that will be used to decrease private payments for such services. The services should be viewed as public goods with externalities and illegal payments which probably hindered access to their consumption will lead to negative consequences.

## Competing interests

The authors declare that they have no competing interests.

## Authors’ contributions

OO and KH conceived the study, participated in the design, data collection and performed the statistical analysis. BU participated in the design, data collection and analysis. OO drafted the manuscript. All the authors read and approved the final manuscript.
